# Training in dual diagnosis interventions (the COMO Study): Randomised controlled trial

**DOI:** 10.1186/1471-244X-8-12

**Published:** 2008-02-27

**Authors:** Elizabeth Hughes, Shamil Wanigaratne, Kevin Gournay, Sonia Johnson, Graham Thornicroft, Emily Finch, Jane Marshall, Neil Smith

**Affiliations:** 1 Health Service and Population Research Department, Institute of Psychiatry, King's College London, Denmark Hill, London SE5 8AF UK; 2 Centre for Clinical and Academic Workforce Innovation, University of Lincoln, Floor 2, Mill 3, Pleasley Vale Business Park, Mansfield, Nottinghamshire, NG19 8RL UK; 3 Department of Mental Health, University College London, Wolfson Building, 48, Riding House Street, London W1W 7EY UK; 4 Addiction Sciences, Institute of Psychiatry, King's College London, Denmark Hill, London SE5 8AF UK

## Abstract

**Background:**

Despite the high prevalence of co-morbid substance use among mental health service users (dual diagnosis), very few mental health workers in the UK have had training and/or clinical experience to equip them to deliver targeted interventions to this client group.

**Method:**

In a randomised controlled trial of training for dual diagnosis interventions, 79 case managers from 12 community mental health teams in South London were randomly allocated to either receive training and follow-up supervision (experimental group) or no training and supervision (control group). Baseline measures of attitude, self-efficacy and knowledge were collected prior to randomisation, and were repeated at 18 months post-training. An intention to treat analysis of follow-up data (adjusted for baseline score for that outcome and team) was performed.

**Results:**

At 18 months post-training, the AAPPQ (The Alcohol and Alcohol Problems Perception Questionnaire) total score was did not differ significantly between the two groups (adjusted difference 7.43 [95% CI -0.86 to 15.71], p = 0.08). There were significant differences in favour of the experimental group on 2 of the 6 subscales of the AAPPQ: 'adequacy of knowledge and skills in working with alcohol" (adjusted difference 3.598 [95% CI 1.03 to 6.16], p = 0.007) and "self-esteem in working with alcohol" (adjusted difference 3.00 [95% CI 0.46 to 5.54], p = 0.021). In addition there were significant improvements for the experimental group on "Knowledge About Dual Diagnosis" (adjusted difference 2.00 [95% CI 0.80 to 3.22], p = 0.002) and "Self-Efficacy Scale" (adjusted difference 13.55 [95% CI 8.00 to 26.86], p = 0.001). The effect of membership of teams was added to the analysis of covariance and this changed the results for only one variable: "self-esteem working with drinkers" was no longer significant.

**Conclusion:**

A brief training course in dual diagnosis interventions had a significant effect on secondary measures of knowledge and self-efficacy that was detectable at 18 months post-training. Improvements in attitudes towards working with drinkers and drug users in mental health settings failed to reach statistical significance. Future research should explore the effects of dose of dual diagnosis training, and the successful integration of skills gained into routine care.

**Trial Registration:**

ISRCTN98891022 14^th ^March 2007

## Background

Developing better services for people with co-morbid mental health and substance use problems has become a national priority in the UK [1]. In 2002, the Department of Health published the Good Practice Guidelines for working with dual diagnosis patients [2]. The guidance acknowledges that co-morbid substance use problems are common within mental health service users and therefore it is "usual rather than exceptional" (p.19) to encounter this clinical problem. Prevalence studies [3-5] have indicated that around a third of people with psychosis have co morbid substance use problems.

There is an emerging evidence base that an integrated approach that combines mental health and substance use approaches (such as cognitive behavioural therapy and motivational interviewing) can be beneficial for people with serious mental health and substance use problems [6-9]. Therefore it was proposed that mental health services should take a primary role in delivering effective integrated interventions for people with serious mental illness and substance use problems [2]. This is known as "mainstreaming". However there is concern as to whether the current mental health workforce is capable of providing such care. A number of surveys of mental health staff reveal a deficit in clinical experience and training in substance use problems [10-12]. One possible solution to this discrepancy between the needs of the service users and the capabilities of the workforce is to develop and deliver specific training programmes that would aim to improve the care that people with dual diagnosis receive.

There is some evidence to suggest that training may be an effective strategy for disseminating psychosocial interventions into the general mental health workforce [13] and Gray et al [14,15] demonstrated in a randomised controlled trial that a 10 day training course in medication management for community psychiatric nurses resulted in increased skill acquisition and improved psychopathology for the service users who received the intervention. Medication management is a combination of motivational interviewing, psycho-education, problem-solving and cognitive behavioural techniques and is designed for people with serious mental illness.

To date the only published study of dual diagnosis training is the COMPASS project in Birmingham. Using a quasi-experimental design, Graham and colleagues evaluated the impact of a 6 half day course of dual diagnosis which was delivered to assertive outreach teams using a whole team training approach [16]. They trained 5 teams in total; 3 teams initially and then the last 2, 18 months later. In addition to the training, there was a manual and a specialist dual diagnosis worker was attached to the team to provide advice, supervision and support in the implementation of the approach. Overall there was a significant improvement in knowledge and confidence in dual diagnosis interventions from pre training to 18 months after training for all five teams. However, there was no significant difference when the teams that had received the training were compared to those who had not had the training. Graham and colleagues [16] recommend that further research should be conducted into the effects of training on both case managers and service users.

The aim of the COMO (co-morbidity) dual diagnosis study is to asses the impact of training in integrated dual diagnosis interventions on case managers working in community mental health teams. A 5 day training course was designed specifically for this study. A separate paper [17] reports the design, methodology, and main service user outcomes. This paper will focus on the training aspect of the study and the impact of this upon case managers.

The main hypotheses tested were that case managers who received the training would have significant improvements in (i) attitudes, (ii) knowledge, and (iii) confidence (self-efficacy) in working with people with dual diagnosis problems in a community mental health setting (when compared to those without training).

## Method

In terms of the case managers, the study was a randomised controlled trial that compared training against no training. This took place in the South London and Maudsley NHS Trust (now Foundation Trust), located in inner London, and was open to all community mental health teams in the organisation. Ethical approval was granted by the Institute of Psychiatry Ethics Committee (now joint South London and Maudsley and Institute of Psychiatry NHS Research Ethics Committee) and was obtained on 17^th ^September, 1999 (reference 075/99). Case managers within the recruited teams were randomly allocated to either training or no training. Informed consent was obtained from the case managers to participate in the trial. They were informed that all aspects of the trial were voluntary, and they could withdraw at any time.

Case managers who were randomised to the control group were offered the training once the follow-up data had been collected at 18 months post-training. To be eligible for inclusion, participants were expected to be working with their caseloads continuously throughout the 18 month research period.

The sample size was calculated for the broader study on the basis of three service user outcomes using p = 0.05 and 80% power. The largest sample size of the three was adopted (220 patients). In terms of case managers, we assumed that 20% of the community mental health case-loads would be people with co-morbid psychosis and substance misuse. Therefore from an average case-load of 20 we would expect to identify 4 service users per case manager, so that if we aimed for a service user sample of 220, we would need to recruit 55 case managers. The number of case managers actually recruited was 79 which was large enough to detect a standardised effect of 0.65 on the Knowledge about Dual Diagnosis (KADD) questionnaire.

Baseline data were collected from participating staff within those teams prior to knowledge of whether they had been randomly allocated to the training group. In order to minimise bias, the research assistants were blind to randomisation, and the randomisation itself was performed by an independent statistician to maintain concealment.

### Primary Outcome Measure

There are to date no valid and reliable measures of attitudes specifically for working with people with co-morbid mental health and substance use. The Alcohol and Alcohol Problems Perception Questionnaire (AAPPQ) [18] was chosen as it has demonstrated reliability and validity in measuring attitudes towards working with alcohol drinkers by generic workers (including mental health workers). It consists of a series of statements about aspects of working with drinkers. Each statement is rated on a 7 point scale from strongly disagree to strongly agree. In this study, the AAPPQ items were repeated, replacing "drinkers" with "drug users" to obtain a measure of attitudes towards drug users.

There are 6 subscales to the AAPPQ: the agents willingness to work with drinkers/drug users (motivation); their self-reported adequacy in knowledge and skills in working with drinkers/drug users, their self-esteem in working with drinkers/drug users, the extent to which they have the right to work with drinkers/drug users (role legitimacy); expectations of job satisfaction in working with drinkers/drug users; and role support in working with drinkers/drug users. Cartwright and colleagues [18] reported that there was good test re-test reliability, and Cronbach alpha coefficients for the scales ranged between 0.7 and 0.9. It has been used in previous studies as a measure of attitudes of mental health workers towards working with drinkers. Lightfoot and Orford [19] used the AAPPQ with community mental health nurses and social workers. They reported an overall Cronbach alpha of 0.83 with the range for the subscales of between 0.7 and 0.9. In this study, good internal consistency was demonstrated; the items for the overall AAPPQ scale had a Cronbach alpha coefficient of 0.9, and the subscales ranged from 0.7– 0.9.

### Secondary Outcome Measures

Basic information about professional background, grade, length of time in profession, previous study relevant to dual diagnosis, and previous clinical experience in substance use field was collected from each participant. The secondary outcomes were measured by scales designed specifically for the study and are therefore intended for exploratory data analyses to generate hypotheses.

The Self-Efficacy Scale (SES) Dual Diagnosis Attitudes (DDA) and the Knowledge about Dual Diagnosis Questionnaires (KADD) were developed for the study as a literature search revealed that dual diagnosis specific measures of knowledge and confidence did not exist at that time. These measures were devised by an expert group with experience in working with and training mental health workers in dual diagnosis interventions. The group agreed that the final versions of the SES, the DDA, and the KADD had face and content validity. The measures were administered to 3 dual diagnosis experts (independent of the study) and they all scored highly on the scales.

The SES consisted of a list of key skills which related to working with someone with mental health and substance use problems. This included assessment, health education, and interventions such as motivational interviewing techniques. Each item was rated from 0% which indicated no confidence at all in being able to perform the specific skill, to 100% (totally confident in ones ability). It had a good internal consistency with a Cronbach alpha coefficient of 0.97, and a range of inter-item correlations from 0.537–1.000. Due to the high internal consistency of this scale future work should assess whether the scale could be refined by using a smaller subset of items.

The DDA consisted of a series of statements about dual diagnosis and each item was rated on a four point scale of 1 disagree strongly to 4 agree strongly. There was no neutral option in order to polarise the views. It had a low internal consistency with a Cronbach alpha coefficient of 0.58. The KADD was a 20 item multiple choice questionnaire based on the content of the dual diagnosis training. Trainees were asked to choose the correct response from a choice of 4. The KADD also had a good internal consistency (Cronbach alpha of 0.70).

In addition to the previously mentioned scales, job satisfaction and burn-out were measured by using the Minnesota Job Satisfaction Scale (MSS) [20], and the Maslach Burn-Out Inventory (MBI) [21]. Burn-out and job dissatisfaction were measured as it has been suggested that trainees are less likely to implement training if they are feeling burnt-out and unhappy in their work. The MSS is a valid and reliable tool that consists of 20 items, and it assesses aspects of work that are a source of satisfaction or dissatisfaction. Each item is assessed on a 5 point scale ranging from 1 (very dissatisfied) to 5 (very satisfied). A score of 60 indicates a neutral level of job satisfaction, and a score over 80 indicates job satisfaction. The MBI is a valid and reliable tool designed to assess burn-out in people whose occupations involve the delivery of care. It consists of 22 items and respondents are asked to rate how often they experience a range of positive and negative work-related feelings, using a scale ranging from 0 (never experienced job related feelings) to 7 (experiencing job related feelings on a daily basis). Three sub scores are derived from the responses: depersonalisation (feeling emotionally detached and callous), emotional exhaustion (feeling emotionally drained and fatigued), and lack of personal accomplishment (feeling that no matter what they do nothing makes a difference; feeling de-skilled). Maslach and Jackson [21] calculated the range of scores for burn-out for different professionals. For mental health workers, high burn-out is indicted by a score of 8 or more on depersonalisation; 21 or more for emotional exhaustion and 28 or less for personal accomplishment. Average burn-out for the subscales falls within the following ranges: depersonalisation 5–7; emotional exhaustion 14–20; and personal accomplishment 33–29.

### The Training Intervention

The training package was designed to increase mental health case managers' skills and competencies to detect, assess, and intervene with co-morbid substance use problems. The expectation was that case managers would integrate the key skills from the training into their practice, so that they could begin to address substance use. In addition to individual work, the training also addressed issues of referral to other agencies (such as specialist substance use services) or joint working with other professionals if the case was complex. The training course was too brief to produce "experts" in dual diagnosis interventions; the aim (within the limits of time and resources) was to help case managers be more effective in working with co-morbid substance use issues.

The training was based on the Institute of Psychiatry, Kings College London, 12 day accredited dual diagnosis module (which was the first accredited clinical skills course in dual diagnosis interventions in the UK), and comprised of 5 days of classroom based training, delivered one day per week, and an 80 page treatment manual. The training utilised a range of methods including didactic presentations, small group discussion and exercises, clinical case discussions, reflection on practice, and role-play. The teaching methods were designed to link theory to the case managers' previous experience, knowledge and clinical experience. After the training had ended, the trainees were offered monthly one hour supervision sessions for the length of the 18 month period post training. The content of the training included the Integrated Treatment Approach [22] which included comprehensive assessment, step-wise working (using Osher and Kofoed's Four Stage Model) [23], taking a flexible and long term view of working with dual diagnosis, Motivational Interviewing techniques (such assessing readiness to change and working with ambivalence) [24], and cognitive behavioural techniques for psychosis including assessment of problem areas and relapse prevention [25].

For consistency, the training and supervision were provided by the person who developed the training package (EH). The skill components of the training were demonstrated by video clips, and also described in detail within the manual. Trainees practiced the skills within the training, and were required to try out the skills in clinical practice and discuss progress within the classroom and in follow-up supervision.

### Statistical Analysis

The strength of evidence for any differences between the two groups was assessed using analysis of covariance with baseline scores as covariate, and post treatment scores as dependent variable. Data were input to databases using SPSS and analysed using SPSS 14 [26] and analysis was performed on all participants who had been originally randomised to receive training or waiting list control, and who had follow-up data. Outcomes were analysed irrespective of whether or not the case manager had participated in or completed the training (intention to treat). Statistical significance was evaluated at p = 0.05 level. No adjustment was made for multiple testing as the secondary outcomes were exploratory in nature. In order to address whether membership of different teams affected the results, the analysis of covariance was repeated with "team" as an additional fixed factor, and the results were compared with the first analysis. In addition, analyses were repeated including any baseline variables that appeared to characterise loss to follow up.

## Results

### Sample

The demographic profile is typical of community mental health teams in London [27]. At baseline, the intervention and control groups were comparable on demographic characteristics (Table 1).

**Table 1:** Trainee Demographics

**Characteristic**	**Intervention Group** **N = 40**	**Control Group** **N = 39**
Age years: **mean (s.d)**	37 (7.3)	37 (6.2)
Number female, **n (%)**	20 (53)	19 (51)
Number White, **n (%)**	21 (58)	26 (78)
Number mental health nurse, **n (%)**	22 (58)	18 (49)
Number > 5 years in current profession, **n (%)**	22 (59)	25 (69)
Number attended study days relevant to dual diagnosis **n (%)**	24 (63)	25 (68)
Number never worked clinically in substance use services **n (%)**	17 (45)	20 (57)

In terms of previous relevant training and clinical experience, 18% of the intervention group and 15% of the control group had attended any study days relevant to working with dual diagnosis, and 22% of the intervention group and 25% of the control group had never worked in a substance misuse treatment service.

**Figure 1:**
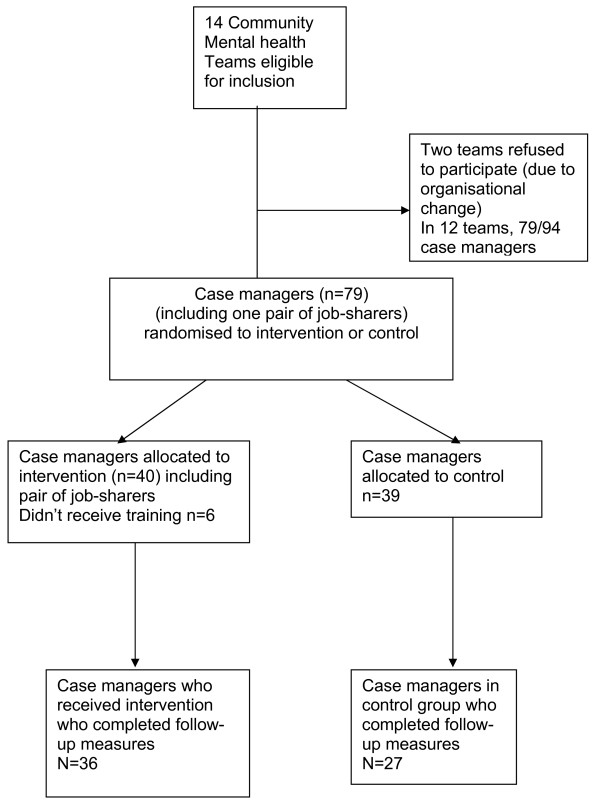
CONSORT Diagram for Case Managers.

In terms of the training, 83% (N = 33) of the 40 case-managers randomised to training participated in it. (See Figure 1 for staff CONSORT diagram [28]). Those who could not attend the training (N = 6) for reasons of illness and annual leave had an individual session with the trainer to go over the course content, attended the follow-on supervision and received the training manual. Of those who did attend the training, 81% attended at least 4.5 days out of 5 days. With reasonably good levels of exposure to the training in the intervention group, all those who entered the trial were included in the analysis (intention to treat). At 18 months post-training, follow-up data were obtained on 80% (63 of 79) of the case managers who completed baseline measures (control = 27; intervention = 36).

Participants evaluated the course on the last day, and rated the training highly on clinical relevance, content, and presentation; however this may be slightly biased as the people who attended on day 5 may have viewed the course more favourably than those didn't attend.

### Primary outcome: Attitudes

At follow-up, after adjusting for baseline differences using analysis of covariance, the overall attitude score at follow-up between intervention and control groups was not significantly different at p = 0.05 although there was a trend toward significance (intervention mean 116.56 s.d. 21.02 vs. control mean 131.15 s.d. 16.15, p = 0.078). There were, however, significant differences on 2 of the subscales of the AAPPQ: "adequacy of knowledge and skills in working with drinkers" (intervention 31.71, s.d. 4.44 vs. control mean 26.29, s.d.6.88, p < 0.05) and "self esteem in working with drinkers" (intervention mean 29.56, s.d.5.16 vs. control mean 25.64, s.d. 4.74, p = 0.021). (Table 2).

**Table 2:** Primary Outcome Measures (AAPPQ) Baseline & Follow-up Means.

**Measure**	**Intervention group**		**Control group**		**Adjusted difference** **(95% CI)**	**P value**
	**Baseline mean (s.d.)** **N = 39**	**Follow-up mean (s.d.)** **N = 36**	**Baseline mean (s.d.)** **N = 39**	**Follow-up mean (s.d.)** **N = 27**		

**AAPPQ overall attitudes (drinkers)**	123.39 (19.69)	131.15 (16.1)	113.73 (18.33)	116.56 (21.01)	7.43 (-0.86 to 15.72)	0.078
**AAPPQ overall attitudes (drug users)**	114.75 (20.85)	123.08 (16.64)	107.97 (20.21)	113.62 (20.45)	4.27 (-3.09 to 11.64)	0.250
**Motivation to work with drinkers**	25.49 (4.25)	26.03 (3.15)	24.46 (4.90)	23.21 (6.08)	1.93 (-0.37 to 4.24)	0.099
**Motivation to work with drug users**	24.32 (4.49)	25.12 (3.60)	23.40 (5.65)	22.78 (5.78)	1.32 (-0.37 to 4.24)	0.209
**Job satisfaction with drinkers**	23.37 (4.35)	23.25 (3.70)	20.71 (3.86)	20.62 (5.72)	0.37 (-1.56 to 3.00)	0.702
**Job satisfaction with drug users**	21.66 (4.49)	21.43 (3.87)	19.85 (4.58)	20.17 (5.81)	0.09 (-2.12 to 1.93)	0.926
**Knowledge and skills with drinkers**	26.89 (7.08)	31.71 (4.45)	23.60 (7.16)	26.29 (6.88)	3.60 (1.03 to 6.16)	0.007
**Knowledge and skills with drug users**	23.68 (6.98)	28.91 (5.67)	21.80 (7.28)	25.00 (6.68)	2.37 (-0.05 to 4.80)	0.055
**The right to work with-drinkers**	19.39 (3.68)	20.09 (4.33)	19.12 (2.95)	20.69 (2.93)	-0.75 (-2.32 to 0.83)	0.345
**The right to work with drug users**	19.81 (3.90)	19.50 (4.03))	19.00 (3.01)	20.62 (2.90)	-1.01 (-2.59 to 0.56)	0.203
**Role support in working with drinkers**	16.39 (2.94)	16.26 (3.27)	15.00 (2.96)	15.45 (3.09)	0.55 (-1.25 to 2.34)	0.543
**Role support in working with drug users**	16.11 (3.19)	15.94 (3.45)	14.68 (2.96)	15.36 (3.08)	0.24 (-1.57 to 2.06)	0.225
**Self-esteem in working with drinkers**	27.71 (5.87)	29.56 (5.16)	25.57 (5.63)	25.64 (4.74)	3.00 (0.46 to 5.54)	0.021
**Self esteem in working with drug users**	26.18 (6.43)	28.00 (5.70)	24.14 (6.31)	24.93 (4.95)	1.93 (-0.60 to 4.47)	0.005

When repeating the ANCOVAs adding "team" as a fixed factor, significance changed for only one sub-scale. The adjusted mean for sub-scale "self-esteem in working with drinkers" was still higher for the intervention group, but felt short of significance (mean difference -2.67 95% [CI -5.67 to 0.33] p = 0.079).

### Secondary outcomes: Knowledge, Self-efficacy and Dual Diagnosis Attitudes

At follow-up, the secondary outcomes; scores for self efficacy (intervention mean 69.49, s.d. 12.55 vs. control mean 52.08 s.d.21.172, p < 0.001) and knowledge (intervention mean 12.97, s.d.2.83, control mean 10.55, s.d. 3.92, p < 0.001), were significantly higher in the intervention group after adjusting for baseline differences using analysis of covariance (table 3). There was no difference in the means for the Dual Diagnosis Attitudes Questionnaire at follow-up. Again the ANCOVAs were repeated with "team" as a fixed factor. The inclusion of team in the analysis did not affect the results (i.e. whether there was a significant difference or not) for the secondary measures.

**Table 3:** Secondary outcome measures baseline and follow-up scores

**Measure**	**Intervention group**		**Control group**		**Adjusted Difference (95% CI)**	**P value**
	**Baseline**	**Follow-up**	**Baseline**	**Follow-up**		

**Self-efficacy**	51.55 (20.29)	68.45 (12.02)	49.15 (23.35)	51.12 (21.19)	17.38 (7.90 to 26.85)	0.001
**Knowledge**	10.50 (4.08)	12.96 (2.83)	10.63 (3.81)	10.55 (3.920	1.996 (0.78 to 3.22)	0.002
**Dual Diagnosis attitudes**	53.77 (3.90)	54.00 (4.78)	53.39 (4.25)	52.87 (3.40)	0.76 (-1.73 to 3.26)	0.539
**Minnesota Job satisfaction**	69.52 (9.84)	70.30 (9.08)	69.21 (12.32)	69.94 (7.51)	1.65 (-3.14 to 6.44)	0.492
**Maslach-depersonalisation**	7.21 (4.60)	7.16 (4.33)	6.36 (4.32)	8.64 (4.99)	-1.47 (-3.31 to 0.37)	0.116
**Maslach-personal accomplishment**	24.73 (5.34)	24.21 (5.50)	24.05 (5.71)	24.31 (6.23)	-0.65 (-3.26 to 1.96)	0.682
**Maslach-emotional exhaustion**	18.15 (9.63)	20.03 (8.25)	16.83 (8.20)	23.58 (9.37)	-3.77 (-8.07 to 0.54)	0.085

### Burn-Out and Job Satisfaction

The scores for the Maslach Burn-out Inventory revealed study participants were experiencing average levels of burn-out on emotional exhaustion and depersonalisation and high levels of burn-out related to personal accomplishment. The Minnesota Satisfaction Survey scores demonstrated that the case managers were not particularly satisfied in their work. There were no differences in scores on the subscales of the Maslach Burnout Inventory and Minnesota satisfaction scales between the two groups at follow-up.

### Participants lost to follow-up

A higher proportion of those who did not complete follow-up questionnaires were in the control group; 32% of those who completed baseline measures did not complete the follow-up questionnaires compared with only 10% of the intervention group. Data was not available on whether the non-completers simply refused to complete the questionnaires or whether they had left the service and could not be traced. Non-completers were not significantly different from completers in terms of sociodemographic characteristics and baseline means for primary and secondary measures. However there were a difference in depersonalisation scores between completers and non-completers in the control group. This difference fell short of significance (p = 0.065), but the analysis of covariance for the primary and secondary outcomes were repeated using depersonalisation as an added covariate in order to control for any bias that this might have introduced into the main analysis. This did not, however, affect the conclusions.

## Discussion

The aim of this paper is to evaluate the effectiveness of a (relatively brief) training programme on case manager attitudes, confidence and knowledge related to working with dual diagnosis. The results have demonstrated that the training intervention has positive effects on some of the case manager outcomes when compared to the control group, and that these effects are detectable at 18 months post training. The impact of the training was mostly concentrated on self-reported confidence in skills, knowledge and an AAPPQ attitude subscale related to adequacy of knowledge and skills with working with drinkers.

However the training did not appear to have an impact on the other AAPPQ attitudes subscales such as role legitimacy, motivation to work with alcohol and drug users, expectation of job satisfaction and role support. This meant that overall there was no significant difference in AAPPQ total score. There was also no difference between the two groups on mean scores for the dual diagnosis attitude scale. The sample was showing signs of burn-out and was not particularly satisfied with their jobs. This may have affected their willingness to implement an intervention that advocates getting more involved with a complex and challenging service user group. Team membership reduced the significance of the subscale "self-esteem about working with drinkers" but not any of the other results. This suggests that there was no major moderating effect of being a member of a particular team on outcomes.

These findings suggest that the training should be revisited to look at how to increase people's sense of motivation, sense of a right to work with these problems, how to increase perception of job satisfaction, and also look at how supported they are in their roles working with people with dual diagnosis. These aspects together will increase workers therapeutic commitment, which Cartwright and colleagues [18] believes is essential for engagement with treatment.

The positive and significant changes in the secondary measures of self-confidence and knowledge are limited in their interpretation. They were both specifically designed for the study and their psychometric properties are yet to be established. In addition they are both self-report questionnaires, and there were no independent verification of skills at baseline or follow-up. Therefore it is difficult to conclude that these positive changes in self-efficacy translated into actual practice change. It would have been useful to measure the level of competency of the case managers' pre and post training, and assess whether they were able to incorporate what they had learnt into their routine practice.

### Limitations of the study

Follow-up measures were only obtained at 18 months post-training. This means that any initial gains as a result of the training will not have been recorded and may have eroded over time. Therefore it is recommended that future training research incorporates regular follow-up points (immediately after training, then 6 monthly intervals) so that acquisition and erosion can be monitored more accurately.

Apart from the AAPPQ attitudes questionnaire, the Maslach Burnout Inventory and the Minnesota Satisfaction Survey, the other measures (Knowledge, Self-Efficacy and Dual Diagnosis Attitudes) were devised specifically for the trial. Further exploration of the psychometric properties of these measures is essential. In terms of multiple testing there is a danger that some of the tests may yield significant results by chance alone. However, as the secondary outcomes were exploratory in nature, and not definitive conclusions. Therefore significant results were areas for informing further research only, and no adjustment were made for multiple tests.

The training "intervention" comprised of three elements; the five training days, an 80 page treatment manual, and monthly supervision. It is unclear whether all these elements were essential for the staff changes, or whether particular aspects more powerful than others. Future training research should assess the effects of the various aspects of the training intervention on outcomes, as well as dose of training. The medication management training [14,15] showed a positive effect on both participants and service users and was twice as long as the dual diagnosis training.

A further limitation was that there was no data on attendance at the supervision sessions; therefore no exploration of the impact of supervision can be made. Future research should ensure that there is an accurate record of supervision for each participant, and that this can be used to compare the outcomes of those who had a high level of supervision and those who had a low level of supervision.

To reduce the effects of contamination, the intervention group were asked not to discuss the content of the training with the team members that had not received the training (control group). However, it is possible that they may have shared their manuals and discussed elements of the training during clinical discussions at team meetings. The only way to minimise contamination would have been to randomly allocate whole teams to training intervention or control conditions. The difficulty with this design is ensuring that enough teams are recruited as clusters and that the teams are similar enough (in terms of catchment area demographics, team skill-mix, remit, models of care etc) to make comparisons meaningful. However, whole team training has been shown to produce positive outcomes, and could make implementation of a psychosocial intervention more effective [29]. Future research should evaluate the impact of whole team dual diagnosis training.

## Conclusion

The COMO project had the ambitious remit of training mental health staff to use a dual diagnosis treatment approach using a relatively brief training intervention. The results demonstrate that a relatively brief training can have some limited effect on how people perceive their skills, and on improving knowledge. The training made less impact on overall attitude change. This finding has major implications for the current policy of "mainstreaming" care for dual diagnosis. It is unlikely that brief training courses such as this (without other service developments such as specialist dual diagnosis workers providing intensive support and supervision, and multi-agency strategies), will be sufficient to meet the needs of this client group. Further research is needed to look at how trainees change their practice and skills following training, and to what extent they are able to implement the approach with the service users on their caseload. In addition further work should explore the effectiveness of of training in differing mental health services, not just community mental health teams. Given the high prevalence of people with substance misuse problems in assertive outreach crisis intervention, acute psychiatric inpatient units, early intervention in psychosis, and forensic mental health services, it may make sense to focus dual diagnosis training in these services where the interventions advocated (such as flexibility, focus on engagement, comprehensive care and longitudinal view) may be more convergent with their current practices. In addition, there needs to be evaluations of other service models for people with co-morbid substance use and mental health problems such as specialist workers based in community teams, and dedicated dual diagnosis services.

## Competing interests

The author(s) declare that they have no competing interests.

## Authors' contributions

EH is the main author of the paper. She designed and delivered the training package, was involved in the development of the case manager outcome measures, and was on the trial steering group. SW was part of the trial steering group, and was involved in the development of the case manager outcome measures, and in editing the paper. KG was on the trial steering group, and was involved in the development of the training package and the case manager outcome measures. SJ was the principle investigator of the trial and has edited and revised this paper. GT was one of the principle investigators of the trial, and has edited and revised this paper. EF and JM were on the trial steering group, and were involved in the development of the training, the case manager outcome measures. NS was a research worker for the trial, and was involved in the collection and management of the data.

## Pre-publication history

The pre-publication history for this paper can be accessed here:


